# Questioning the recovery of dissociated traumatic memories under psilocybin: comment on “Therapeutic emergence of dissociated traumatic memories during psilocybin treatment for anorexia nervosa”

**DOI:** 10.1186/s40337-025-01484-8

**Published:** 2025-12-04

**Authors:** Samuli Kangaslampi, Max Wolff, Manoj K. Doss, Lilian Kloft-Heller, Henry Otgaar

**Affiliations:** 1 Faculty of Social Sciences/Psychology, Tampere University, Tampere, 33014 Finland; 2 Department of Molecular Neuroimaging, Central Institute of Mental Health, Medical Faculty Mannheim, Heidelberg University, Mannheim, Germany; 3 Department of Psychiatry and Neurosciences, Charité-Universitätsmedizin Berlin, Corporate Member of Freie Universität Berlin and Humboldt-Universität zu Berlin, Campus Charité Mitte, Berlin, Germany; 4 Department of Psychiatry and Behavioral Sciences, Charmaine & Gordon McGill Center for Psychedelic Research and Therapy, The University of Texas at Austin Dell Medical School, Austin, TX USA; 5 Faculty of Psychology and Neuroscience, Maastricht University, Maastricht, The Netherlands; 6 Institute of Psychology, University of Bonn, Bonn, Germany; 7 Faculty of Law and Criminology, KU Leuven, Leuven, Belgium

**Keywords:** Psilocybin, Recovered memory, Trauma, Dissociation, Amnesia, Psychedelics

## Abstract

In their recent case report article, Peck and colleagues suggested that two patients recovered dissociated traumatic memories during psilocybin treatment for anorexia nervosa. These case reports are of clinical and scientific interest and confirm that psychedelics may induce vivid memory-like experiences. However, the reports warrant scrutiny. Here, based on what is known about recovered memories and the effects of psychedelics, we argue that the authors may not have adequately considered alternative explanations. The cases do not necessarily demonstrate that psilocybin induces recovery of dissociated traumatic memories or could treat dissociative amnesia. We further caution against the authors’ suggestion of explicitly preparing patients for the emergence of forgotten material.

In their recent paper, Peck and colleagues [1] present two case reports of individuals who received psilocybin-assisted treatment for anorexia nervosa, during which they experienced the apparent recovery of traumatic memories. What the authors present is certainly of clinical and scientific interest. These cases confirm that psychedelics may induce intense, vivid memory-like experiences judged to be highly meaningful. However, the interpretation that these experiences represent the recovery of genuinely dissociated traumatic events warrants scrutiny, and we wish to provide a critical commentary on the paper.

First, as the authors acknowledge, the occurrence of such vivid memory-like experiences was noted already in the first wave of psychedelic research some seventy years ago. However, already then it was also noted that such experiences may involve “a mixture of facts and fantasy” [2] and can refer to disordered, fragmented, or distorted reconstructions interspersed with symbolic content [3].

The authors indeed refer to the importance of “not guiding [participants] towards confirmation or denial” if a patient reports a memory-like experience under psychedelics. This is in line with therapeutic best practices underscoring the need for caution when interpreting recovered memories, regardless of how real they may feel and how much emotional meaning they may carry [4, 5]. They also note that “the recalled events cannot be confirmed”, that is, there was no possibility for independent corroboration. However, they still explicitly state, including in the article’s title, that in these two cases, there was in fact an “emergence of previously dissociated traumatic memories”, also described as “emergence de novo of previously forgotten, highly meaningful traumatic events”. With such statements, the authors appear to leave little doubt that these experiences involved recovery of accurate (dissociated) memories of past events. However, there are alternative explanations that should be considered, as detailed below.

Second, as the authors concede, dissociative amnesia is subject to controversy. An important caveat is that dissociative amnesia and its related term *repressed memory* are unfalsifiable constructs. They refer to the unconscious storage of traumatic memories. However, it is impossible to test these unconsciously dissociated or repressed memories as people are unaware of them [6, 7]. Furthermore, for many claimed cases of dissociative amnesia there are alternative explanations that have not been adequately considered (e.g., normal forgetting) [8].

Third, when people report that they recovered memories of which they were previously unaware, it is vital to recognize that these recovered memories might be of several different types. False memories are one possibility. It has been amply demonstrated that false recovered memories may develop in therapeutic contexts, especially if therapists believe in the idea (and therapeutic importance) of repressed memories [7, 9]. However, if a person reports they have recovered a memory, that memory may also refer to an authentic experience in their past (i.e., spontaneous recovered memory) [10]. One relevant possibility for such spontaneous recovered memory is that an adult may come to understand that what happened to them was abuse and reappraise events that they did remember, but did not earlier consider as emotionally negative or traumatic. This reinterpretation can be misinterpreted as not having been aware of the memory before [11]. People may also believe they have not remembered a traumatic event before, even when they have demonstrably discussed them years earlier [12]. Clearly, even if people are able to remember an event, they may avoid or try to suppress the memory, or otherwise not think about it for years. Further, even if psychedelics might aid retrieval of memories based on real experiences, memory experiences under psychedelics could still be partially inaccurate - for example, the result of fusing of elements from (perhaps several) memories and other sources. Thus, even if the memory-like experience is about an event that did occur, there is no guarantee that all the details recalled correspond with the facts of what happened.

To be clear, we are not claiming that the presented cases did not involve accurate memories of a traumatic event being recalled and processed under psychedelics. However, we believe that the authors do not show that this is indeed what happened, nor that these memories were previously dissociated (or repressed), despite calling what took place “emergence of previously dissociated traumatic memories”. For experiencing therapeutic benefits, the literal truth of recovered memories may not always matter. But to avoid harms and the possibility of false or inaccurate memories, beliefs or accusations emerging, as well as to keep the scientific record straight, it is key to carefully consider alternative explanations in cases like those they present. This is where the authors may not have been critical enough in how they present their cases. As Peck and colleagues agree, clinicians and researchers should indeed refrain from confirming (or dismissing) the accuracy of reported memory experiences if they do not have outside corroborating information. While confirmation is problematic, dismissal or overt skepticism is also not appropriate and may cause harm by, e.g., disrupting the therapeutic relationship.

Fourth, the particular effects of psychedelics on memory may further complicate this matter. Overall, the effects of psychedelics on the recall and experiencing of autobiographical memories are not well understood. There is evidence that they may make memory-like experiences especially vivid and strengthen a sense of re-living [13]. However, despite anecdotal claims, it has never been demonstrated that psychedelics could objectively improve veridical access to memories that would otherwise be inaccessible. If anything, the reductions in default mode network functional connectivity cited by the authors and the prominent hippocampal 5-HT_2A_ expression on inhibitory neurons would be expected to impair memory retrieval [14, 15]. Consistent with these points, one study in rodents found psilocin to impair the retrieval of a previously formed hippocampal-dependent memory, an effect that persisted following acute drug effects, suggesting that the original memory trace was altered by attempting to retrieve it under acute effects [16]. In fact, many psychoactive drugs, including the psychedelic-like drug MDMA, can produce false memories when attempting to remember during the acute drug effect [17, 18]. Simultaneously, psychedelics may enhance suggestibility [19] and tend to imbue experiences under their influences with a *noetic quality*, an undeniable sense of knowledge [20]. As a result, whatever insights, beliefs or apparent memories that transpire under the influence of psychedelics may be experienced as particularly convincing, real, and meaningful, regardless of their veracity or source [21]. One study found that such noeticism associated with a psychedelic experience can subsequently be misattributed, resulting in memory distortions [22].

Fifth, based on these cases, in their Discussion, the authors tentatively suggest potential for psychedelic treatment of dissociative amnesia. We would approach this notion with great caution. More broadly, acknowledging that Peck and colleagues are not suggesting this, we would certainly caution against reviving any therapeutic framework aimed at recovering dissociated traumatic memories, particularly in altered states of consciousness. While psychedelics may offer valuable opportunities for meaning-making and therapeutic insight, their clinical use must be grounded in critical awareness of memory’s reconstructive nature. The history of recovered-memory therapy, including methods like deep regression, hypnosis, and sodium amytal interviews, provides disconcerting examples of the formation of false memories, psychological harms, and misplaced blame [7, 9].

Psychedelics may have interesting effects on autobiographical recall that warrant careful study. Their potential for treating trauma-related disorders, where memory-related mechanisms, such as enhanced extinction learning, may well be involved [23], should also be explored (but note that psychedelics may also enhance fear conditioning, which may speak to the initial destabilization following dosing in the first patient). However, there is no evidence so far that psychedelics can truly recover dissociated traumatic memories or treat dissociative amnesia.

Finally, in preparatory sessions for the clinical use of psychedelics, the authors suggest “preparing and informing a person for the potential of emerging historical autobiographical material […] Specifically, it may be ethically necessary to inform participants of the possibility of forgotten or unfamiliar historical material emerging. At the same time, this should be done carefully so as not prime an expectation, assign undue influence to this possibility, or inadvertently insinuate therapeutic value to such an occurrence.” [1] In contrast, we believe it would be more prudent to inform patients about the potential noetic quality of psychedelic experiences, including memory-related ones, clarifying that subjective feelings of “revealed truth” are common but should not be mistaken as evidence for objective truth. Based on this understanding, it should also be agreed by both patients and clinicians beforehand that any insights or memory-like experiences will not be directly confirmed or denied in subsequent integration work but (as recommended by Peck and colleagues, too) instead discussed from a neutral stance, allowing patients to arrive at their own appropriate level of conviction or skepticism.

## Data Availability

No datasets were generated or analysed during the current study.
